# Integrated Analysis of Prognostic Genes Associated With Ischemia–Reperfusion Injury in Renal Transplantation

**DOI:** 10.3389/fimmu.2021.747020

**Published:** 2021-09-07

**Authors:** Di Zhang, Yicun Wang, Song Zeng, Min Zhang, Xin Zhang, Yuxuan Wang, Zijian Zhang, Xi Wang, Xiaopeng Hu

**Affiliations:** ^1^Department of Urology, Beijing Chaoyang Hospital, Capital Medical University, Beijing, China; ^2^Institute of Urology, Capital Medical University, Beijing, China; ^3^Department of Immunology, School of Basic Medical Sciences, Advanced Innovation Center for Human Brain Protection, Beijing Key Laboratory for Cancer Invasion and Metastasis, Capital Medical University, Beijing, China; ^4^Department of Oncology, Capital Medical University, Beijing, China; ^5^Beijing Key Laboratory of Emerging Infectious Diseases, Institute of Infectious Diseases, Beijing Ditan Hospital, Capital Medical University, Beijing, China

**Keywords:** ischemia–reperfusion injury, renal transplantation, graft survival, immune cell, NF-κB signaling

## Abstract

**Background:**

Ischemia–reperfusion injury (IRI) remains an inevitable and major challenge in renal transplantation. The current study aims to obtain deep insights into underlying mechanisms and seek prognostic genes as potential therapeutic targets for renal IRI (RIRI).

**Methods:**

After systematically screening the Gene Expression Omnibus (GEO) database, we collected gene expression profiles of over 1,000 specimens from 11 independent cohorts. Differentially expressed genes (DEGs) were identified by comparing allograft kidney biopsies taken before and after reperfusion in the discovery cohort and further validated in another two independent transplant cohorts. Then, graft survival analysis and immune cell analysis of DEGs were performed in another independent renal transplant cohort with long-term follow-ups to further screen out prognostic genes. Cell type and time course analyses were performed for investigating the expression pattern of prognostic genes in more dimensions utilizing a mouse RIRI model. Finally, two novel genes firstly identified in RIRI were verified in the mouse model and comprehensively analyzed to investigate potential mechanisms.

**Results:**

Twenty DEGs upregulated in the process of RIRI throughout different donor types (living donors, cardiac and brain death donors) were successfully identified and validated. Among them, upregulation of 10 genes was associated with poor long-term allograft outcomes and exhibited strong correlations with prognostic immune cells, like macrophages. Furthermore, certain genes were found to be only differentially expressed in specific cell types and remained with high expression levels even months after RIRI in the mouse model, which processed the potential to serve as therapeutic targets. Importantly, two newly identified genes in RIRI, *Btg2* and *Rhob*, were successfully confirmed in the mouse model and found to have strong connections with NF-κB signaling.

**Conclusions:**

We successfully identified and validated 10 IRI-associated prognostic genes in renal transplantation across different donor types, and two novel genes with crucial roles in RIRI were recognized for the first time. Our findings offered promising potential therapeutic targets for RIRI in renal transplantation.

## Introduction

Ischemia–reperfusion injury (IRI), a major and inevitable complication that occurred during organ transplantation, involves an initial restriction of blood supply followed by the subsequent restoration of perfusion (1). In the field of renal transplantation, IRI remains a leading cause of acute kidney injury (AKI), contributing to an increased risk of in-hospital mortality and poor long-term outcomes (2, 3). Moreover, renal IRI (RIRI) after transplantation elicits cascades of pathological conditions which enhance allograft immunogenicity and primarily stimulate alloimmune responses, eventually leading to acute rejection and progression to chronic allograft nephropathy (4, 5).

During the process of IRI, hypoxic injury following production of reactive oxygen species (ROS), due to reoxygenation, results in profound inflammatory responses and different forms of cell death-like apoptosis and ferroptosis (6–8). Current experimental strategies to prevent or alleviate RIRI can be focused on scavenging ROS, reducing inflammation, or promoting cell survival and regeneration, including cellular therapy, pharmacological treatment, and ischemic preconditioning (9–12). Despite those methods, there is still a lack of effective treatment in clinical settings (13). Besides, with the expansion of donor pools in recent years, donation after brain death (DBD) and donation after cardiac death (DCD) have been gradually increased. However, these deceased allografts experienced more severe IRI and were at higher risk of graft loss (14, 15). Therefore, it is of great potential and importance to search for novel and valid therapies for RIRI regardless of donor types to improve clinical outcomes.

With the rapid advancement of genome-wide gene expression analysis, remarkable progress has been made in understanding the molecular complexity of individual variability in clinical settings (16). At present, numerous studies have been performed on IRI gene expression profiles, and potential genes were investigated in the pathogenesis and progression of IRI (17, 18). However, due to the heterogeneity among each cohort, current results had a lot of differences and were hard to translate. Therefore, integrated analysis of multiple datasets is needed to break the limitations and reduce the false-positive results of single-cohort studies and therefore enable the identification of reliable therapeutic molecular targets (19). In this study, gene expression profiles of IRI specimens from four renal transplantation cohorts were collected to seek robust RIRI-associated prognostic genes. Due to limitations of access to human tissues, we utilized mouse RIRI datasets to perform time-course and specific cell-type analyses, thus enriching our understanding of these genes in the occurrence and progression of RIRI. In brief, the current study identified and comprehensively analyzed novel genes that would contribute to precisely treating IRI and improving the prognosis of renal transplant patients.

## Materials and Methods

### Data Collection and Preprocessing

As summarized in **Table 1**, we systematically collected 11 datasets containing gene expression profiles of specimens from the Gene Expression Omnibus (GEO) database. Detailed information about the operations and sample size of all available cohorts is shown in **Supplementary Tables 1** and **2**. Human IRI-associated biopsies were taken before and after organ implantation and used to compare the transcriptomic differences pre- and post-reperfusion. Microarray datasets were normalized through “limma” package (20), while the normalization of RNA sequencing datasets was carried out by the “DESeq2” package (21). Log_2_ transformation was performed for all datasets.

**Table 1:** Information of 11 datasets included in this study.

Datasets	Platforms	Species	Tissues	Sample size	Applications	References (PMID)
GSE43974	Illumina HumanHT-12 V4.0 Expression BeadChip	Homo sapiens	Kidney	260	Discovery of DEGs	25427168
GSE126805	Illumina HiSeq 3000	Homo sapiens	Kidney	82	Validation of DEGs	30429361
GSE90861	Illumina NextSeq 500	Homo sapiens	Kidney	46	Validation of DEGs	30094915
GSE21374	Affymetrix Human Genome U133 Plus 2.0 Array	Homo sapiens	Kidney	282	Graft survival analysis	20501945
GSE52004	Affymetrix Mouse Gene 1.0 ST Array	Mus musculus	Kidney	45	Cell type analysis	24569379
GSE98622	Illumina HiSeq 2000 and Illumina NextSeq 500	Mus musculus	Kidney	40	Time course analysis	28931758
GSE151648	Illumina HiSeq 3000	Homo sapiens	Liver	80	Cross-organ investigation	32426849
GSE23649	Illumina HumanHT-12 V3.0 Expression BeadChip	Homo sapiens	Liver	66	Cross-organ investigation	No publication
GSE12720	Affymetrix Human Genome U133 Plus 2.0 Array	Homo sapiens	Liver	42	Cross-organ investigation	19353763
GSE127003	Affymetrix Human Genome U133 Plus 2.0 Array	Homo sapiens	Lung	92	Cross-organ investigation	32060066
GSE18995	Affymetrix Human Genome U133 Plus 2.0 Array	Homo sapiens	Lung	35	Cross-organ investigation	No publication

### Study Design

**Figure 1:**
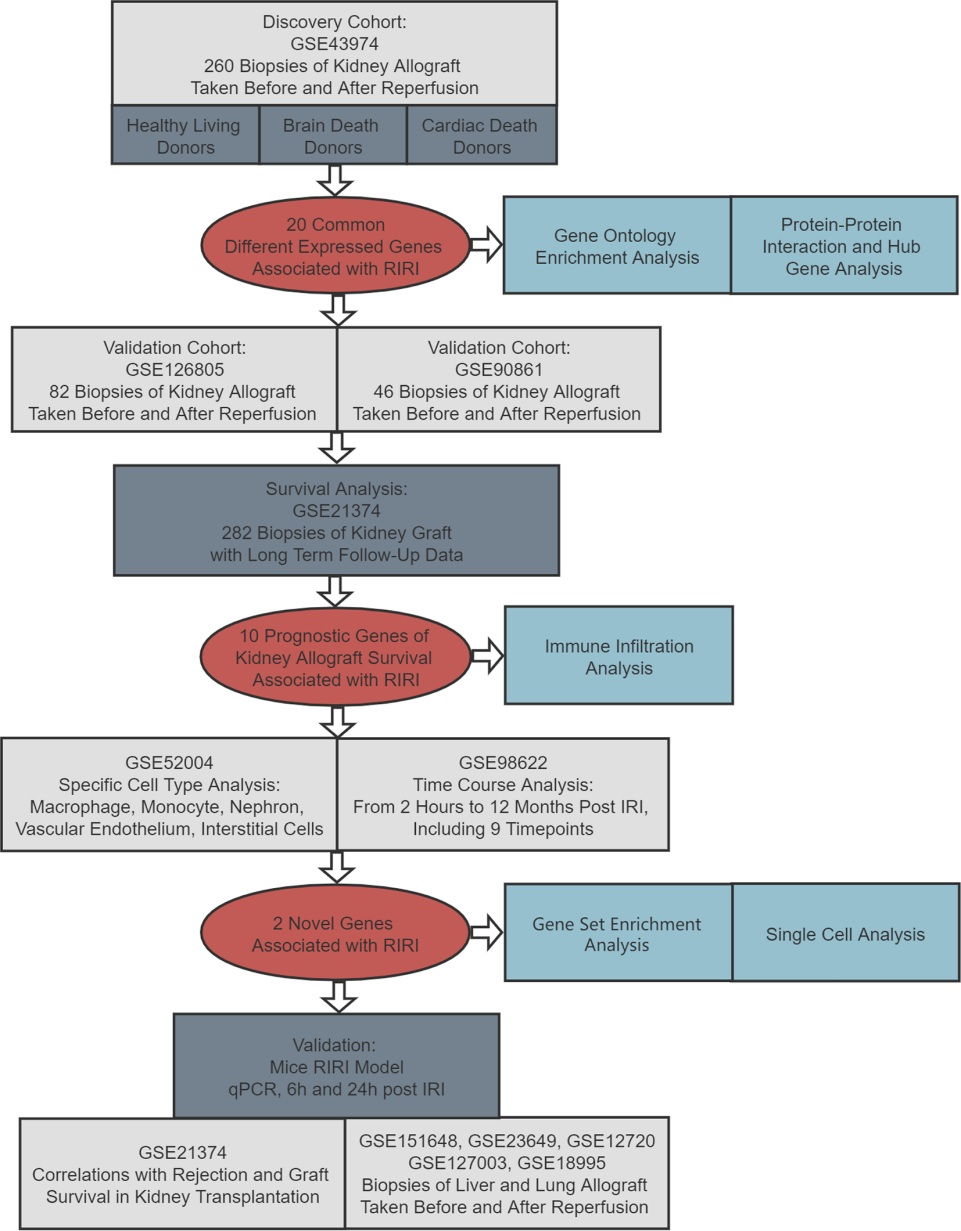
Flowchart of this study. RIRI: renal ischemia–reperfusion injury, IRI: ischemia–reperfusion injury, qPCR: quantitative polymerase chain reaction.

The flowchart of the current article is presented in **Figure 1**. In the discovery cohort, 260 biopsies taken before and after reperfusion in renal allografts acquired from healthy living donors, brain-dead donors, and cardiac death donors were included to seek common DEGs associated with RIRI across donor types (17). Those DEGs were utilized for functional analysis and then verified in another two independent renal transplant cohorts, consisting of 82 and 46 eligible biopsies, respectively (22, 23). Subsequently, we performed survival analysis to further screen prognostic DEGs utilizing 282 biopsies of kidney allografts with long-term follow-up data (24), and the time–space expression patterns of prognostic DEGs were also detected in specific cells and at various time points after RIRI in mouse model (18, 25). After systematic literature searching and reviewing, two newly identified genes in RIRI were selected for further analysis. Their expression levels were validated by quantitative real-time polymerase chain reaction (qRT-PCR) in the mouse RIRI model. Moreover, their roles in renal and other solid organ transplantations (liver and lung) were explored (26–28). Additionally, we conducted gene set enrichment and single-cell analyses for these novel genes.

### Identification and Validation of DEGs in RIRI

Differential expression analyses were performed to identify DEGs before and after reperfusion. We compared samples pre- and post-reperfusion because previous studies reported that there was little difference of transcriptome before and after cold ischemia and no pathway was enriched based on DEGs, while the transcriptome changes were significant after reperfusion (17). The threshold was set as the adjusted *p*-value < 0.05 and the absolute value of log_2_-fold change > 1. DEGs observed simultaneously in all three kinds of donor types (DBD, DCD, and donation from living donors) were selected for validation and subsequent analyses.

### PPI Analysis and Functional Annotations of DEGs

Candidate DEGs were submitted to the STRING online database (https://string-db.org/) for protein–protein interaction (PPI) analysis (29), and the PPI network was visualized by Cytoscape software (30). Hub genes were then identified through CytoHubba, a plugin in Cytoscape. Subsequently, Metascape (https://metascape.org/gp/index.html#/main/step1), a powerful annotation analysis tool functioning by integrating several authoritative data resources (GO and KEGG were applied as annotations), was applied to analyze the potential biological processes and signaling pathways occurring in the episodes of IRI (31). The threshold was set as an adjusted *p*-value < 0.05, a minimum enrichment score of 2, and a minimum overlap of five genes.

### Graft Survival and Immune Infiltrate Analysis

To further explore the effects of DEGs on long-term allograft survival, the GSE21374 dataset was applied, which contains gene expression and graft survival data of renal transplant patients with 51 graft loss and 231 graft survival. ImmuCellAI (http://bioinfo.life.hust.edu.cn/ImmuCellAI#!/), a novel gene expression-based tool, was used to estimate the abundance of 24 immune cells in renal biopsies (32). Subsequently, we applied univariate Cox regression analyses to assess the prognostic significance of DEGs and immune cells; their predictive abilities were also quantified by bootstrapped C-index. Correlations between gene expression and immune cell infiltration were also calculated by Pearson analysis, and the threshold was set as an absolute value of coefficient > 0.25 and *p*-value<0.05.

### Specific Cell Type and Time Course Analyses

In GSE52004, expression levels of candidate prognostic DEGs in macrophage, monocyte, nephron, vascular endothelium, and interstitial cells of renal tissues taken from bilateral RIRI mice (three mice in each group) were acquired. Besides, RNA-seq data of renal tissues collected at 10 different time points (three mice in each group) following RIRI—2 and 4 h; 1, 2, and 3 days; 1, 2, and 4 weeks; and 6 and 12 months—were obtained in GSE98622. Combining the above two datasets, time–space expression patterns of prognostic genes were elucidated.

### Animals and Procedures

In animal experiments, C57BL/6 mice (8–10 weeks old, male) were purchased from Vital River Laboratory Animal Technology (Beijing, China). All animals were maintained in a specific pathogen-free facility at the Medical Research Center. Mice were divided into three groups (sham group, IRI_6h group, and IRI_24h group), each group consisting of six mice. Both renal pedicles were clamped for 45 min in the IRI group, and kidney samples were harvested at 6 and 24 h after reperfusion. Mice in the sham group underwent all surgical procedures except bilateral renal pedicle occlusion.

### Assessment of Kidney Injury and Quantification of mRNA Expression

Kidney injury was assessed by measuring levels of serum creatinine (SCr) and blood urea nitrogen (BUN). Blood samples were collected from the vena cava, and the serum was separated by centrifugation at 3,000 rpm for 15 min and then sent to the Department of Biochemistry to detect levels of Scr and BUN. Besides, renal samples were fixed in 4% formaldehyde, dehydrated, and embedded in paraffin. Tissue sections (4 mm) were stained with hematoxylin–eosin (HE). Total RNA was extracted using TRIzol reagent (Invitrogen, Carlsbad, CA), and reverse transcription reactions were performed following instructions. The levels of transcripts were determined by qRT-PCR using a standard protocol from the SYBR Green PCR kit (Toyobo, Osaka, Japan). Primers of *Rhob* and *Btg2* are listed in **Supplementary Table 3**.

### Further Exploration of Novel Genes

We firstly performed Kaplan–Meier (K-M) survival analysis to show the prognostic value of two novel RIRI genes for renal allograft long-term survival and compared their expression levels between biopsy-proven rejection and non-rejection allograft samples. Next, IRI specimens taken from liver and lung transplantations were utilized to investigate their potential roles in other solid organ transplantations. In addition, gene set enrichment analysis (GSEA) of these two genes was conducted using post-reperfusion samples in GSE43974 based on hallmark and cell-type signature gene sets from the molecular signatures database (MSigDB) by the “clusterProfiler” R package. Finally, single-cell analysis was performed using the Human Protein Atlas (https://www.proteinatlas.org/).

### Statistical Analysis

All statistical analyses were carried out by R software (version 3.6). The D’Agostino and Pearson omnibus normality test was performed to determine if data follow a normal distribution in each comparison. If the data passed the normality test, parametric tests (two-tailed unpaired t-tests, one-way ANOVA with Tukey’s correction for multiple comparisons, and Pearson correlation) would be conducted. On the contrary, non-parametric tests were applied (Mann–Whitney-U test, one-way ANOVA using Kruskal–Wallis with Dunn’s correction for multiple comparisons, and Spearman correlation). The reported results were all considered statistically significant at the 5% critical level (*p* < 0.05).

## Results

### Identification of DEGs in RIRI

**Figure 2:**
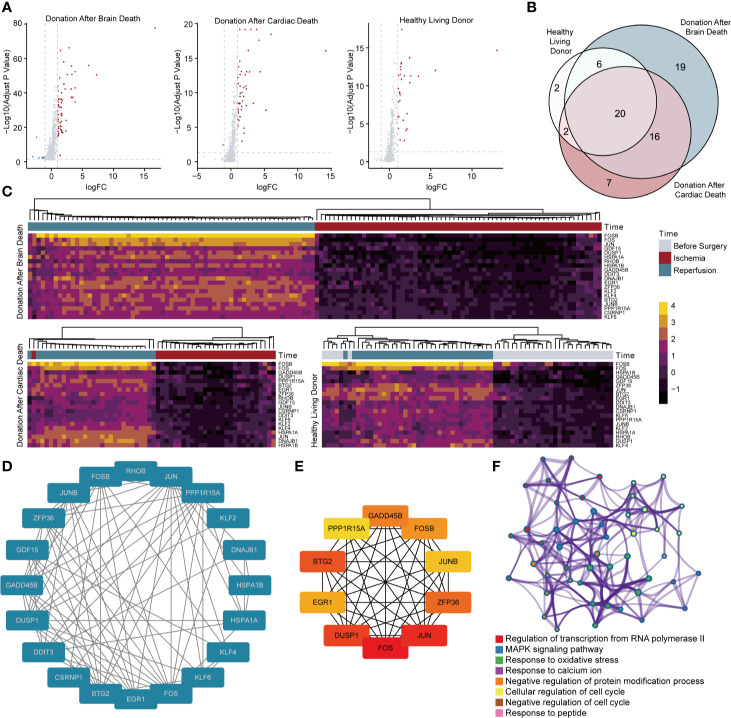
Screening and analysis of DEGs for RIRI. **(A)** Volcano plots show DEGs between samples taken before and after reperfusion in brain death (61 upregulated and 9 downregulated genes), cardiac death (45 upregulated and 1 downregulated genes), and healthy living donors (30 upregulated genes), respectively. **(B)** The Venn diagram demonstrates the intersection of DEGs among three kinds of donor types. **(C)** Clustering heat maps illustrate expression levels of 20 robust DEGs in different phases of renal transplantation. Protein–protein interaction network of 20 DEGs **(D)** and 10 hub genes **(E)**. The deeper color indicates higher connectivity. **(F)** Network of enriched clusters, where each node represents one statistically significant term of biological process, and terms with similarity of more than 0.3 are connected by edges. DEGs, differentially expressed genes.

Differential expression analyses were performed in biopsies taken before and after reperfusion from living and deceased donor kidneys (DBD and DCD) to select robust and general genes. As illustrated in **Figure 2A**, there were 70 DEGs (61 upregulated genes and 9 downregulated genes) and 46 DEGs (45 upregulated genes and 1 downregulated gene) observed in DBD and DCD donor samples, respectively, while 30 DEGs (30 upregulated genes) were identified in renal tissues derived from healthy living donors (**Supplementary Table 4**). Twenty DEGs simultaneously selected in three types of donations were all upregulated and then included for subsequent analyses (**Figure 2B**). Heat maps of DEGs were depicted in **Figure 2C**, and hierarchical clustering analysis showed that DEGs ideally clustered samples before and after IRI.

### PPI Network and Functional Enrichment Analysis

Using the STRING database, 20 upregulated DEGs were constructed into the PPI network (**Figure 2D**). Results showed that 20 genes were all strongly connected without exception. Among them, 10 genes, including *BTG2*, *EGR1*, *DUSP1*, *FOS*, *JUN*, *ZFP36*, *JUNB*, *FOSB*, *GADD45B*, and *PPP1R15A*, were identified as hub genes in accordance with the degree score generated by CytoHubba and showed an even closer network (**Figure 2E**). Metascape analysis illustrated clusters of enriched biological processes (**Figure 2F**). Results manifested that identified genes were significantly enriched in regulation of transcription from RNA polymerase II, MAPK signaling pathway, response to oxidative stress, and multiple processes related to the cell cycle.

### Validation of DEGs in RIRI

**Figure 3:**
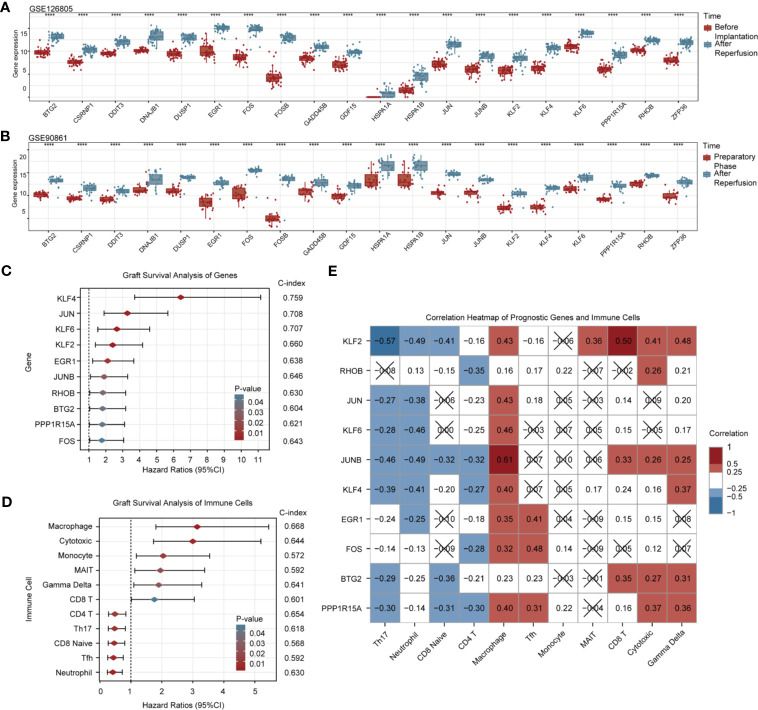
Validation and graft survival analysis of DEGs. Box and scatter plots compare the expression levels of 20 upregulated RIRI-associated DEGs **(A, B)**. Forest plots show hazard ratios and their 95% confidence intervals of 10 prognostic DEGs **(C)** and immune cells **(D)**. C-indexes are also displayed on the right side of both pictures. **(E)** Correlation heat map for gene expression and immune cell infiltration; red and blue represent positive and negative correlations, respectively. No statistically significant correlations (*p* > 0.05) are denoted by black crosses.

To verify the robustness of 20 upregulated DEGs in RIRI, we employed two independent renal transplantation cohorts. As demonstrated in **Figures 3A, B**, those DEGs all remained differentially expressed after reperfusion in renal allograft tissues suffered from IRI.

### Graft Survival Analysis of DEGs and Immune Cells

Among 20 DEGs, 10 of them were associated with renal allograft survival (**Figure 3C**, **Supplementary Table 5**). Higher expressions of *KLF4*, *JUN*, *KLF6*, *KLF2*, *EGR1*, *JUNB*, *RHOB*, *BTG2*, *PPP1R15A*, and *FOS* in renal allograft tissues indicated poorer prognosis. The C-indexes of the top 3 genes with the highest hazard ratio (*KLF4*, *JUN*, *KLF6*) were above 0.7, and C-indexes of all prognostic genes were above 0.6. By evaluating the infiltration of immune cells, high infiltration of macrophage, cytotoxic T cell, monocyte, MAIT, γδT, and CD8^+^ T cell were risk factors for graft survival, while CD4^+^ T cell, Th17 cell, CD8^+^ naive cell, Tfh, and neutrophil were protective factors. The C-indexes of prognostic immune cells were around 0.6 (**Figure 3D**, **Supplementary Table 6**).

In addition, correlations between genes and immune cells with prognostic values are shown in **Figure 3E**. Prognostic genes were almost negatively correlated with subsets of protective immune cells and exhibited partly positive correlations with potential risk immune cells, which were in accordance with survival analysis results. Notably, macrophages showed strong correlations with nearly all risk genes.

### Specific Cell Type and Time Course Analyses of Prognostic Genes

**Figure 4:**
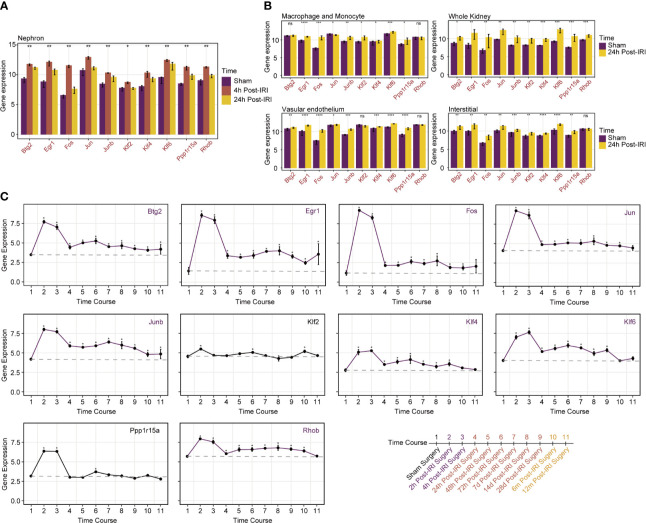
Specific cell type and time course analyses of prognostic genes in mice RIRI model. **(A)** Histograms represent expression levels of prognostic genes in the nephron of mice from sham and 4- and 24-h post-IRI groups. **(B)** Histograms compare gene expression levels between sham and 24-h post-IRI samples of macrophage and monocyte, vascular endothelium and interstitial, and whole kidney. *p < 0.05; **p < 0.01; ***p < 0.001; ****p < 0.0001; ns, not statistically significant. **(C)** Line plots with 95% confidence intervals show changes in expression levels of prognostic genes over time post-IRI; a total of 10 different time points were included in analyses as illustrated on the bottom-right side of the picture. Genes marked in purple kept higher expressed than baseline until 12 months after IRI.

As for gene expression changes in specific cell types during the episodes of RIRI, the above 10 prognostic genes all remained upregulated in whole kidney tissues 24 h after IRI, which suggested the strong concordance of RIRI-invoked genes between the human cohort and the mouse model. Similarly, these 10 genes were all upregulated in the nephron (**Figure 4A**), while certain genes showed no changes of expression levels in the vascular endothelium, interstitium, macrophage, and monocyte (**Figure 4B**). Among them, *Rhob* was found to be only upregulated in the nephron, which suggested its special role in specific cells.

As illustrated in **Figure 4C**, all genes showed similar upregulated changes in 24 h post-IRI surgery. Intriguingly, over a time series after 24 h, the expression of genes included *Btg2*, *Egr1*, *Fos*, *Jun*, *Junb*, *Klf4*, *Klf6*, and *Rhob* remained higher than the baseline level stably until 12 months after IRI. In contrast, minor changes around the baseline were detected after 24 h post-IRI surgery for other genes (*Klf2* and *Ppp1r15a*). Those alterations indicated that the continuously upregulated genes like Btg2 and Rhob may cause long-term injury for renal allografts and therefore were suitable for therapeutic targets.

### Verification of Novel Genes in a Mouse Model

**Figure 5:**
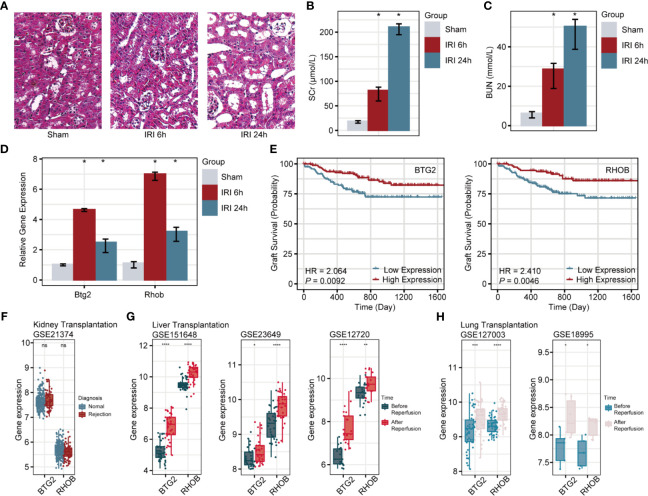
Verification of *Btg2* and *Rhob* in a mouse model and comprehensive analysis in clinical cohorts. HE staining of renal tissues from sham and 6-h post-IRI, and 24-h post-IRI groups **(A)**. Histograms illustrate differences in SCr **(B)**, BUN **(C)**, *Btg2*, and *Rhob* expression **(D)** among sham, 6-h post-IRI, and 24-h post-IRI groups. **(E)** K-M survival curves for *Btg2* and *Rhob*. **(F)** Expression levels of *Btg2* and *Rhob* between normal and rejection groups. Differential expression patterns of *Btg2* and *Rhob* before and after reperfusion in liver **(G)** and lung transplantations **(H)**. *p < 0.05; **p < 0.01; ***p < 0.001; ****p < 0.0001; ns, not statistically significant. HE, hematoxylin–eosin; SCr, serum creatinine; BUN, blood urea nitrogen.

Among 10 prognosis-related DEGs, *Btg2* and *Rhob* were firstly identified to be associated with RIRI, so they were chosen to be confirmed in the RIRI mouse model. The results of HE staining, Scr, and BUN are shown in **Figures 5A–C**. Through expression validation by qRT-PCR, *Btg2* and *Rhob* were validated to be upregulated 6 and 24 h post-IRI in the mouse model (**Figure 5D**).

### Further Exploration of *BTG2* and *RHOB*

In GSE21374, specimens with a higher expression of *BTG2* (HR = 2.064, *p* = 0.0092) and *RHOB* (HR = 2.41, *p* = 0.0046) showed poorer allograft survival in renal transplant patients (**Figure 5E**). However, there were no differences in their expression levels between normal and rejection samples (**Figure 5F**). Similar to the upregulated changes after IRI in renal biopsies, *BTG2* and *RHOB* displayed the same trend during the response to ischemia/reperfusion in liver (**Figure 5G**) and lung transplantations (**Figure 5H**).

**Figure 6:**
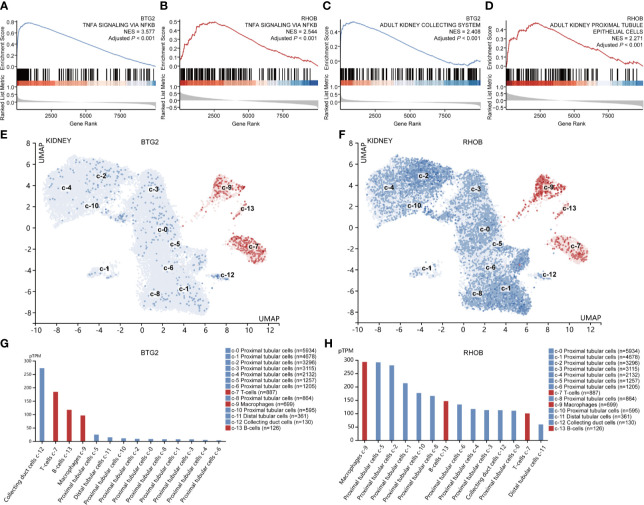
Gene set enrichment and single-cell analyses of *Btg2* and *Rhob*. Enriched hallmark gene signatures **(A, B)** and cell type signatures **(C, D)** of *Btg2* and *Rhob*. UMAP clustering of *Btg2* and *Rhob* in the kidney **(E, F)**. The red color represents immune cell types, and the blue color represents other renal cell types. Cell types are sorted from high to low according to expression levels in bar graphs **(G, H)**. UMAP, uniform manifold approximation and projection.

Interestingly, the top enriched biological pathways of *BTG2* and *RHOB* in IRI kidney samples were both “TNFα signaling *via* NF-κB” (**Figures 6A, B**, **Supplementary Table 7**). When using cell-type gene signatures as reference, the top enriched cell types in the kidney of *BTG2* and *RHOB* were “collecting system” and “proximal tubule epithelial cells,” respectively (**Figures 6C, D**, **Supplementary Table 8**). The results of single-gene GSEA were confirmed by the single-cell analysis, which showed that *BTG2* was most highly expressed in collecting duct cells and *RHOB* was most highly expressed in proximal tubular cells (**Figures 6E–H**).

## Discussion

In recent years, IRI has attracted increasing attention especially in the field of transplantation due to the extension of the donor pool by employing kidneys from DBD and DCD donors (14, 15). Compared to healthy living donors, kidneys from DBD donors are at a higher risk of organ damage and inferior graft survival because of the pathological changes during brain death (33), while kidneys from DCD donors suffer from prolonged warm ischemia times during cardiac arrest which leads to an increased risk of delayed graft function (34). Therefore, three types of donations were all included in our research, and as a result, 20 common upregulated DEGs regardless of donor types were identified for RIRI. Those DEGs were also successfully validated in another two independent cohorts of renal transplantation, which further demonstrated their stability and robustness. Moreover, those DEGs were found to be strongly connected as a tight network without exception based on the PPI network and mainly enriched in several biological processes such as regulation of transcription and cell cycle, MAPK signaling pathway, response to oxidative stress, and calcium ion. Similarly, the above biological processes were all well elucidated in previous studies (5, 35, 36), proving the reliability of our identified genes.

As a highly relevant detrimental consequence of transplantation, IRI adversely affects clinical outcomes of transplantation patients (5, 37, 38). Therefore, we investigated the impact of IRI-associated genes on long-term graft survival. The results of survival analyses demonstrated that the upregulation of 10 genes (*KLF4*, *JUN*, *KLF6*, *KLF2*, *EGR1*, *JUNB*, *RHOB*, *BTG2*, *PPP1R15A*, and *FOS*) was significantly associated with poor long-term allograft outcomes. Among these genes, *KLF2*, *KLF4*, and *KLF6* are highly conserved zinc finger transcription factors that regulate cell apoptosis, proliferation, differentiation, and migration. *KLF2* and *KLF4* are proved to be highly expressed in the endothelium during the episodes of IRI and responsible for disease progression (39, 40), while *KLF6* targeting improved IRI-induced AKI through effects on inflammation, apoptosis, and renal function (41). Combined with our results, those prognostic genes may control the progression of RIRI to adversely affect long-term outcomes of posttransplant patients and would be possibly utilized as effective targets, which was also applicable for other confirmed prognostic genes in the current study.

RIRI engages the innate and adaptive immune responses and interplays with cytokine generation within the kidney, resulting in tissue damage (42). Among 24 immune cells estimated by ImmuCellAI, aberrantly high infiltrations of macrophages, cytotoxic T cells, monocytes, MAIT, γδT, and CD8^+^ T cells were harmful to patients after renal transplantation, while CD4^+^ T cells, Th17 cells, CD8^+^ naive cells, Tfh, and neutrophils were protective. As the leading risk factor, macrophages were expected to have a significant function in immune-mediated kidney injury since these cells function as both effector cells and antigen-presenting cells, thereby connecting the innate and adaptive immune systems (43). Their influx upon reperfusion of the post-ischemic kidney seemed to facilitate the inflammatory cascade and contribute to the development of renal fibrosis (43, 44). Their relationships with expression levels of prognostic genes may serve as potential regulatory pathways. For example, expression levels of *JUNB* were positively correlated with amounts of macrophages, which meant that *JUNB* may stimulate the activation of macrophages or the infiltration of macrophages facilitates the expression of *JUNB* thus participating in downstream responses. Some other immune cells that negatively impact clinical outcomes like γδT cells, which were proved to be protective after being deficient (45), deserved more investigation in further research and may be targeted to reduce amounts, thus improving prognosis. Notably, the administration of cells to modulate the course of IRI has attracted considerable interest in recent years (9). In particular, mesenchymal stem cells and regulatory T cells have been reported as promising therapies to reduce renal IRI in animal models (46, 47). That is to say, those protective immune cells detected in this study would be possibly used as supplementations to alleviate renal IRI for future exploration.

To deepen our understanding of the identified key RIRI genes, we further employed two studies based on the mouse RIRI model, which offers more dimensions of sampling. We firstly validated expression levels of 10 prognostic genes to ensure that similar changes could also be found in animal models. Results illustrated consistent upregulated trends in both whole kidney tissues and nephrons after bilateral renal pedicles clipped for 28 min followed by 24-h reperfusion. Our data suggest a high concordance between human and mouse IRI datasets, indicating that multiple injury-invoked gene regulatory responses are conserved across species, as previous studies reported (22). Besides, genes like *Rhob*, which was found to be upregulated only in the nephron, may perform functions as a response to IRI in specific cell types, thus deserving further experiments. These findings may contribute to filling up the vacancies of organ-wide approaches which have not elucidated distinct cell-type responses involved in the pathophysiology of RIRI. As for time-course analysis, several genes including *Btg2* and *Rhob* remained more highly expressed than baseline for 12-month post-IRI surgery, further indicating their long-term effects on allografts.

Among prognostic genes identified in our research, it is worth noting that *Btg2* (B-cell translocation gene 2) and *Rhob* (Ras homolog family member B) were hardly investigated in RIRI. Therefore, we verified the aberrant upregulated expression of these two genes in our mouse RIRI model. Moreover, although a higher expression of *Btg2* and *Rhob* indicated poorer prognosis, there was no difference in their expression levels between normal and rejection groups. That is to say, the mechanisms underlying the prognostic value of these two IRI-associated genes were not related to rejection, which ruled out the impact of transplant-related factors. It is also worth mentioning that *Btg2* and *Rhob* kept being more highly expressed after IRI in liver and lung transplantations. These similar transcriptional alterations across organs further indicated their promising potential in the field of solid organ transplantation across organ types.

*BTG2*, a p53-inducible gene, exerts its antiproliferation effects by regulating cell-cycle progression, apoptosis, and differentiation (48). As for *RHOB*, belonging to the Ras superfamily, it could stimulate multiple pathways that regulate gene transcription and then control the growth and differentiation of cells (49). In our research, GSEA indicated that NF-κB signaling was the most significantly enriched pathway of both *Btg2 and Rhob*. Previous studies reported that the NF-κB signaling pathway was activated in RIRI, leading to aggravation of tubular injury and exacerbation of inflammatory response, while its inhibition improves renal function (50, 51). Besides, *Btg2* can be aberrantly stimulated by activation of NF-κB signaling as a response to oxidative stress (52), and *Rhob* is transiently upregulated by cell exposure to inflammatory cytokines, which may be dependent on the NF-κB pathway (53). Combined with our results, we infer that *Btg2* and *Rhob* function through the NF-κB signaling pathway during RIRI. In addition, highly consistent results of GSEA and single-cell analysis revealed that *Btg2* and *Rhob* were widely expressed in kidney tissues and especially most highly expressed in collecting duct cells and proximal tubule cells, respectively. These results lay the foundation for future experiments, guiding the specific pathway and cell type. Overall, these novel prognostic genes may function through NF-κB signaling in specific cell populations during RIRI and were suitable for precisely targeted in future therapeutic studies.

Our research successfully identified and validated 10 RIRI-associated genes, which have significant prognostic impacts on renal allograft survival. Cell type and time course analyses in the mouse RIRI model additionally enhanced our understanding of disease progression over space and time. Moreover, *Btg2* and *Rhob* were identified to be involved in the process of RIRI for the first time and processed the potential to be applied to other solid organs (as shown in the liver and lung). Our results not only set the foundation for mechanisms investigation but also offered novel and promising therapeutic targets for RIRI in clinical practice.

## Data Availability Statement

The datasets presented in this study can be found in online repositories. The names of the repository/repositories and accession number(s) can be found in the article/**Supplementary Material**.

## Ethics Statement

The animal study was reviewed and approved by the Ethics Committee of Beijing Chaoyang Hospital.

## Author Contributions

XH, XW, DZ, and YicW participated in the research design. DZ carried out the statistical analysis. SZ, MZ, XZ, YuxW, and ZZ conducted the animal experiments. DZ drew the figures, and YicW drafted the manuscript; XH, XW, DZ, and YicW participated in modifying the manuscript. All authors contributed to the article and approved the submitted version.

## Funding

This work was supported by the General Program of the National Natural Science Foundation of China (NSFC) (81970645, 82000711) and the joint fund of the Beijing Municipal Commission of Education and Natural Science Foundation of Beijing Municipality (KZ202010025036).

## Conflict of Interest

The authors declare that the research was conducted in the absence of any commercial or financial relationships that could be construed as a potential conflict of interest.

## Publisher’s Note

All claims expressed in this article are solely those of the authors and do not necessarily represent those of their affiliated organizations, or those of the publisher, the editors and the reviewers. Any product that may be evaluated in this article, or claim that may be made by its manufacturer, is not guaranteed or endorsed by the publisher.
