# Supplementary figures and images for: Integrated Analysis of Prognostic Genes Associated With Ischemia–Reperfusion Injury in Renal Transplantation
Source: Front Immunol. 2021 Sep 7;12:747020. doi: 10.3389/fimmu.2021.747020 (PMC8452995; doi:10.3389/fimmu.2021.747020)

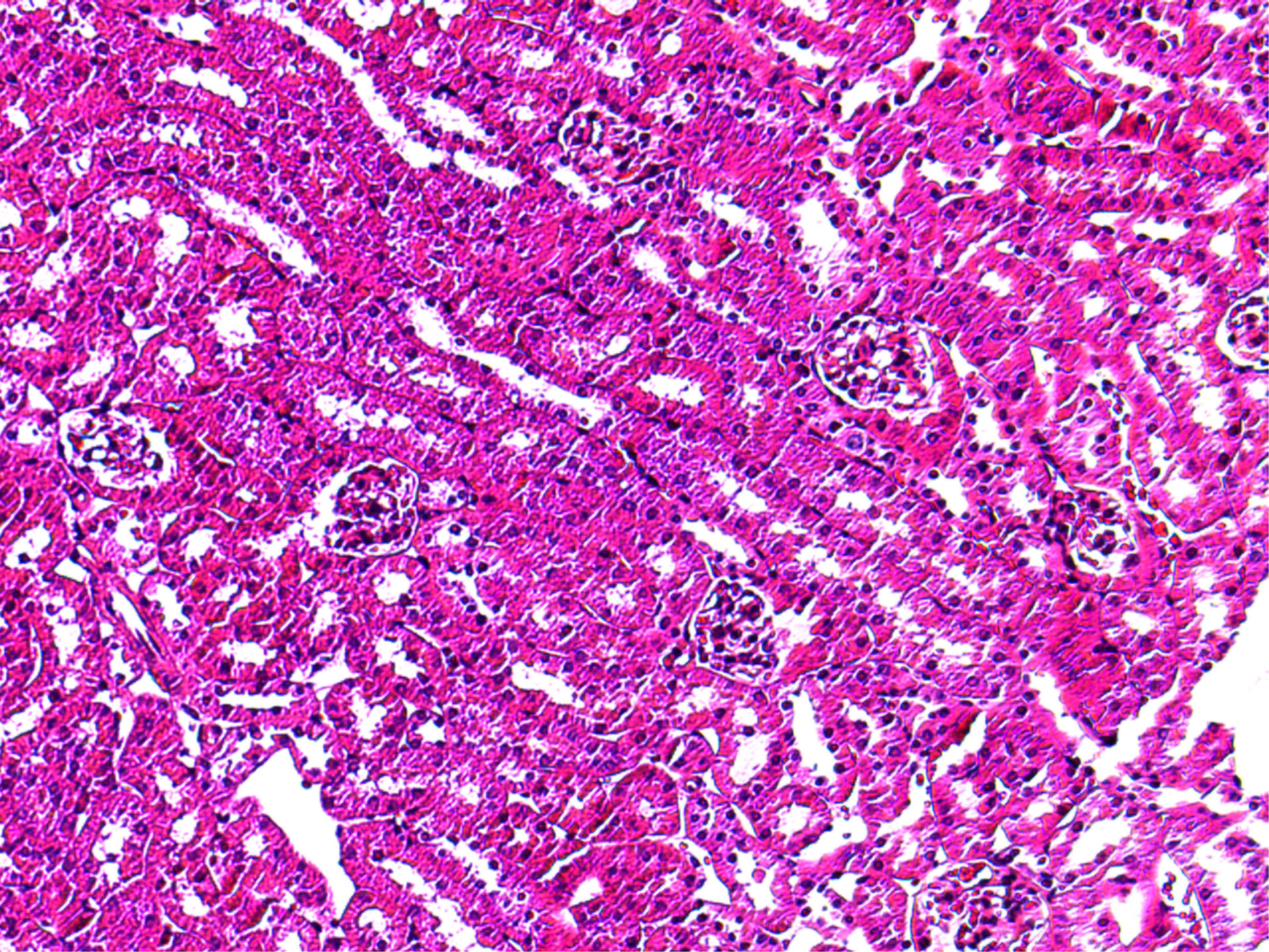

Supplement: Supplementary file 2 [file Image_1.tif]

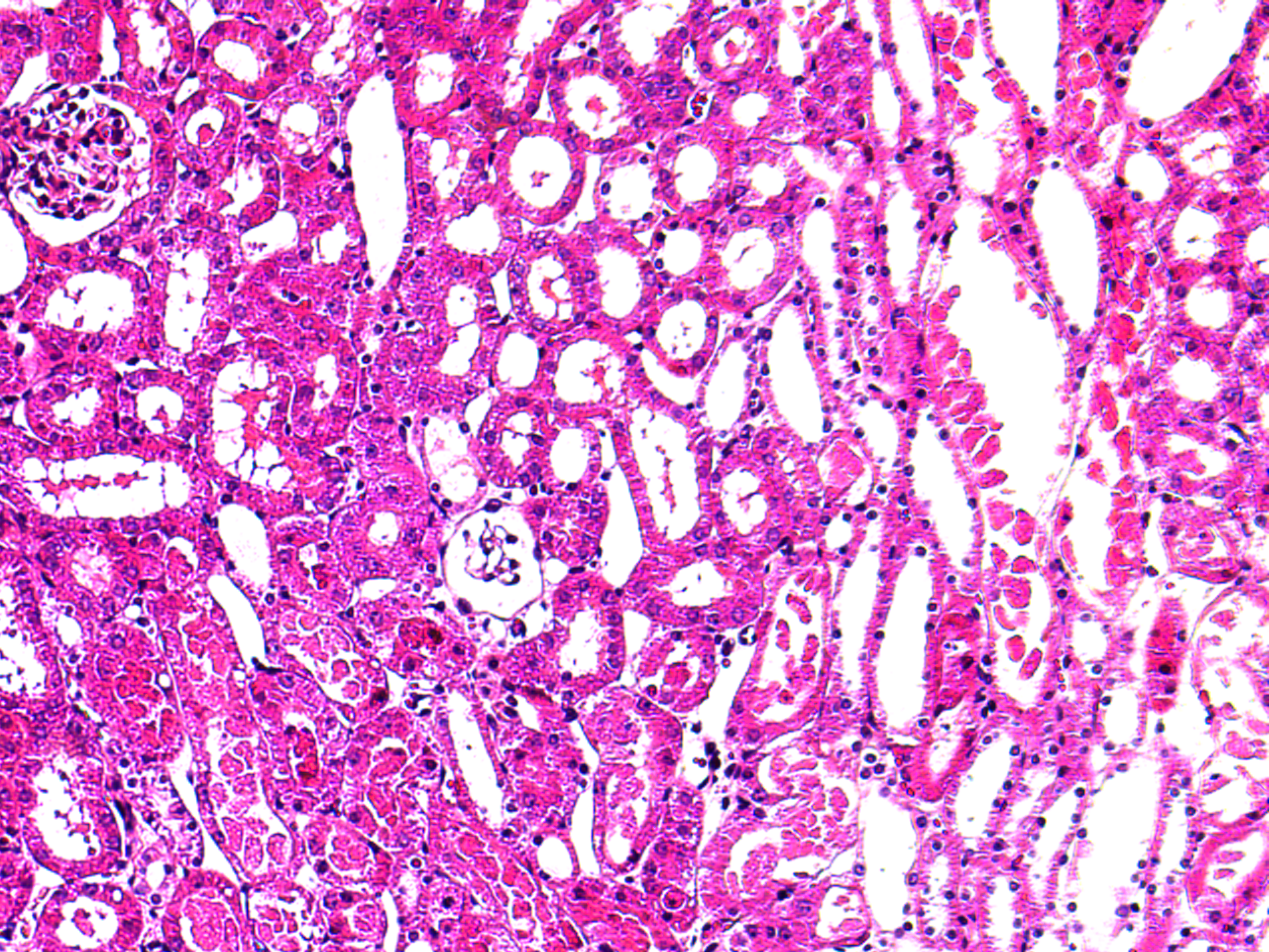

Supplement: Supplementary file 3 [file Image_2.tif]

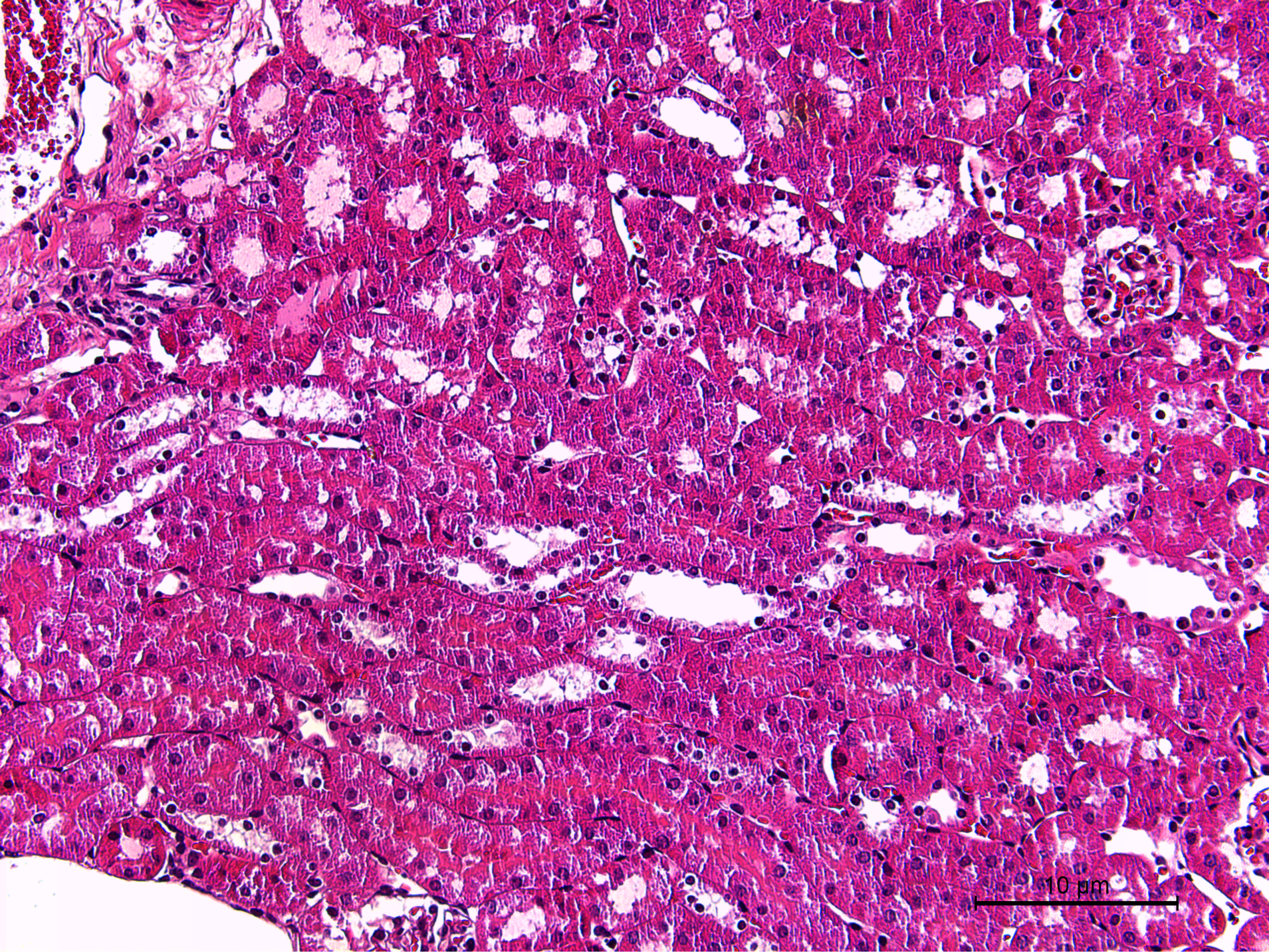

Supplement: Supplementary file 4 [file Image_3.tif]
